# Sample Size Requirements for Popular Classification Algorithms in Tabular Clinical Data: Empirical Study

**DOI:** 10.2196/60231

**Published:** 2024-12-17

**Authors:** Scott Silvey, Jinze Liu

**Affiliations:** 1 Department of Biostatistics School of Public Health Virginia Commonwealth University Richmond, VA United States

**Keywords:** medical informatics, machine learning, sample size, research design, decision trees, classification algorithm, clinical research, learning-curve analysis, analysis, analyses, guidelines, ML, decision making, algorithm, curve analysis, dataset

## Abstract

**Background:**

The performance of a classification algorithm eventually reaches a point of diminishing returns, where the additional sample added does not improve the results. Thus, there is a need to determine an optimal sample size that maximizes performance while accounting for computational burden or budgetary concerns.

**Objective:**

This study aimed to determine optimal sample sizes and the relationships between sample size and dataset-level characteristics over a variety of binary classification algorithms.

**Methods:**

A total of 16 large open-source datasets were collected, each containing a binary clinical outcome. Furthermore, 4 machine learning algorithms were assessed: XGBoost (XGB), random forest (RF), logistic regression (LR), and neural networks (NNs). For each dataset, the cross-validated area under the curve (AUC) was calculated at increasing sample sizes, and learning curves were fit. Sample sizes needed to reach the observed full–dataset AUC minus 2 points (0.02) were calculated from the fitted learning curves and compared across the datasets and algorithms. Dataset–level characteristics, minority class proportion, full–dataset AUC, number of features, type of features, and degree of nonlinearity were examined. Negative binomial regression models were used to quantify relationships between these characteristics and expected sample sizes within each algorithm. A total of 4 multivariable models were constructed, which selected the best-fitting combination of dataset–level characteristics.

**Results:**

Among the 16 datasets (full-dataset sample sizes ranging from 70,000-1,000,000), median sample sizes were 9960 (XGB), 3404 (RF), 696 (LR), and 12,298 (NN) to reach AUC stability. For all 4 algorithms, more balanced classes (multiplier: 0.93-0.96 for a 1% increase in minority class proportion) were associated with decreased sample size. Other characteristics varied in importance across algorithms—in general, more features, weaker features, and more complex relationships between the predictors and the response increased expected sample sizes. In multivariable analysis, the top selected predictors were minority class proportion among all 4 algorithms assessed, full–dataset AUC (XGB, RF, and NN), and dataset nonlinearity (XGB, RF, and NN). For LR, the top predictors were minority class proportion, percentage of strong linear features, and number of features. Final multivariable sample size models had high goodness-of-fit, with dataset–level predictors explaining a majority (66.5%-84.5%) of the total deviance in the data among all 4 models.

**Conclusions:**

The sample sizes needed to reach AUC stability among 4 popular classification algorithms vary by dataset and method and are associated with dataset–level characteristics that can be influenced or estimated before the start of a research study.

## Introduction

### Background

Machine learning (ML) is becoming increasingly popular within the domain of health care data analysis and clinical decision-making [1]. The lack of a fixed model specification and distributional assumptions allows for these methods to learn complex relationships that are not necessarily linear in nature, such as high-order interactions and polynomial effects. Due to this, most popular machine-learning algorithms require much larger sample sizes than traditional statistical methods [2]. However, exact amounts are not clear, and there are many different ML algorithms, each containing its own limitations and properties [3]. Furthermore, in traditional statistical analysis, we can often analytically derive equations that measure how the sample size needed to detect a certain prespecified effect will behave under certain assumptions [4]. Due to the data-driven and algorithmic nature of ML methods, which rely on computational approaches rather than statistical theory to capture relationships, an empirical approach is necessary in order to understand the behavior of these methods under varying conditions.

It is known that, for any given dataset, there is a point where adding additional samples will not increase the performance metrics of the model considerably [5]. Thus, it becomes important to collect enough data to optimize these metrics while also accounting for this performance ceiling and the budgetary or computational concerns that may arise when collecting substantial amounts of unnecessary data.

Another reason for the difficulty in selecting a proper sample size when applying ML is the lack of a true end point or common metric of interest. As discussed previously, the traditional target for sample size determination methods is the statistical power to detect a certain effect size [4]. In ML, since predictive performance rather than parameter estimation is usually of interest, this end point becomes unclear. A commonly used metric of predictive performance is prediction accuracy, defined as the proportion of correct classifications made [6]. However, the prediction accuracy is related to the distribution of the outcome; for a rare event, accuracy can be high even with a completely noninformative model [7]. As a result, a fairer performance metric is the area under the receiver operating characteristic curve (area under the curve [AUC]), which evaluates model predictions over a range of probability thresholds from 0 to 1 [8]. AUC is widely used to evaluate the performance of an ML algorithm and has several desirable properties. First is interpretability—a higher AUC indicates a higher degree of separability, and an AUC of 0.5 implies a completely random prediction, while an AUC of 1.0 indicates perfect classification. A second desirable property of AUC is insensitivity to the proportion of cases versus controls in the dataset [9]; because the entire range of probability thresholds is considered, AUC can also be considered the “average” sensitivity (true-positive rate) over all possible values of specificity (true-negative rate). While the AUC is commonly used to evaluate the initial performance of an ML algorithm, other metrics, such as calibration [10], may also be preferred once modeling reaches later stages. However, it is necessary to ensure that a trained ML model can first make stable predictions before assessing further metrics such as calibration or threshold-selection.

### Related Works

The concept of empirically estimating the performance of a classification algorithm as the training set size increases has been widely explored in a variety of different settings. This is typically done by creating a “learning curve,” measuring a metric (such as classification accuracy) as a function of sample size [11]. Perlich et al [12] compared logistic regression (LR) approaches versus decision-tree–based approaches, demonstrating that LR often outperforms tree induction in small samples, but decision trees excel as the sample size becomes large. Mukherjee et al [13] developed a method to assess the error rate of a classifier as a function of sample size using an inverse power-law model. Their method was introduced in the context of DNA microarray data, which often contains a large amount of features and limited access to samples due to cost restraints. Figueroa et al [14] modified the original learning curve fitting process by using nonlinear weighted least squares to favor future predictions, using 3 moderately sized datasets to demonstrate their algorithm. Provost et al [15] used learning curves and efficient progressive sampling to show that classification algorithms eventually converge to a stable accuracy with increasing sample size, mainly focusing on the methodology of the sampling scheme. More recently, van der Ploeg et al [16] used several clinical datasets and a simulation-based approach to show that modern classification algorithms such as neural networks (NNs) and random forest (RF) require at least 200 events per variable to reach a stable AUC. Richter and Khoshgoftaar [17] experimented with learning curves on biomedical big data with limited labels and heavy class imbalance, using 1% of the full dataset AUC as their stopping rule. Because the cost of labeling certain types of data is expensive, it is important to maximize the quality of the data while minimizing costs. They found that a semisupervised approach and pseudolabeled data generated from a small amount of actual data could accurately predict future performance.

### Study Aim

Previous contributions have focused on the methodology of learning curve fitting or estimating future performance from an already-collected sample. In those that have examined similar end points (ie, AUC plateau or stability over a variety of algorithms), the number of real-world datasets included was small, modern gradient boosting techniques (XGBoost [XGB], etc) were not examined because they had not yet been developed, and the impact of dataset–level characteristics on sample sizes was not extensively studied [15,16]. Previous literature has also mostly used small datasets in the context of -omics type data. In general, a focused clinical study often contains fewer features, a wide variety of variable types (ie, numeric, categorical, ordinal, etc), and fewer correlated features than -omics data [18]. This study aims to develop algorithm-specific sample size guidelines using dataset–level variables that can be estimated or manipulated by researchers before any data has been collected, analogous to a sample size calculation performed in a traditional power analysis. The focus of these guidelines was on the stable internal validity of each method, which is typically the first benchmark used to assess performance when attempting to develop a predictive model. We examined 4 popular binary classification algorithms in the context of clinical research, where the aim is to predict a health-related outcome such as a disease state or event. The contributions of this study include a learning curve analysis of 16 real-world datasets, an examination of modern gradient-boosting methods (XGB) within this analysis, and concrete sample size guidelines based on dataset-level characteristics.

## Methods

### Dataset Description

We have collected 16 public-access clinical datasets ranging from sample sizes of 70,000-1,000,000. A detailed description of dataset sources, variables included, and outcomes can be found in Multimedia Appendix 1. It should be noted that 8 of these 16 datasets were artificially created from smaller real-life datasets using Bayesian Network Generation, and their details have been previously discussed [19]. All datasets contained a single binary outcome, such as a disease state, with a combination of continuous numeric, discrete numeric, or binary predictors. Continuous numeric features were considered to have at least 10 unique values.

A detailed description of specific data preprocessing steps can be found in Multimedia Appendix 1. In summary, nominal variables were converted to binary variables based on arbitrary binning rules, and variables containing text values (ie, “gender”: male vs female) were also converted to binary variables. Missing data was present in 3 datasets only (CDC Heart Disease [2022], Diabetes130, and COVID-19), although the amount of missingness was quite low among these sets (1.3%, <1%, and <1%, respectively). Without knowing additional information regarding the nature of these missing values, we considered them missing completely at random (MCAR) and performed mean imputation [20].

### Classification Algorithms

We examined the following binary classifiers on each dataset: Logistic Regression (LR) [21], Random Forest (RF) [22], XGBoost (XGB) [23], and Neural Networks (NNs) [24]. These algorithms were selected due to their widespread and popular use in clinical data analysis [25]. We performed LR by fitting a multivariable model using all predictors without any variable selection or regularization methods. For the RF and XGB algorithms, hyperparameters were left at their default values, which can be found in the R documentation [22,23]. For NNs, we used the R (R Foundation for Statistical Computing) package *h2o* [26] to perform our analyses; we considered one hidden layer with 20 units and 10 epochs of data training. The type of NN used by “h2o” is a multilayer feedforward artificial NN, also known as multilayer perceptron (MLP). The activation function used in the hidden layers was the default linear rectifier, with softmax activation in the final output nodes for probability estimation and classification. Other NN hyperparameters were again left at their default values, which can be found in the documentation [26].

### Learning Curve Approach

From the 16 datasets studied, we evaluated the cross-validated area under the curve (CV-AUC) as a function of increasing sample size. Cross-validation is a method of assessing the internal validity of a classifier; it works by splitting the entire training dataset into *k* folds and fitting *k* models, with a single fold left out for evaluation in each model [27]. The final cross-validated metric (in our case, AUC) is calculated by taking the average performance over all *k* of the held-out folds. As a result, the entire training dataset is used to generate an estimate of out-of-sample performance. The learning curve approach is detailed below:

1. Create a list of proposed training set sizes.
2. At each point in the sample size interval, randomly sample 10 subdatasets of size n from the full dataset.
3. In each of the 10 subdatasets, estimate the (5-fold, outcome-stratified) CV-AUC on the proposed algorithm of choice. Average the ten CV-AUC values to generate an estimate of out-of-sample performance at a given n.
4. Repeat at the next n in the list.

For the first step, the training set size list usually consisted of 10 evenly spaced points ranging from n=500 to n=50,000, but if stability was not reached by n=50,000, the end point was extended. For LR, the final n was lower, as the AUC from these models typically became stable much earlier than more complex ML algorithms. A full description of the sample size intervals used for each dataset and each algorithm can be found in Multimedia Appendix 1 (Table S1). Stability was defined as the smallest n where the CV-AUC was within 2 points (0.02) of the observed full–dataset AUC. For example, if the full–dataset AUC was 0.85, we would obtain the smallest n where a CV-AUC of 0.83 was first surpassed. The full–dataset AUC for each classification algorithm was calculated using 5-fold stratified cross-validation (CV) on the entire dataset. We chose this stopping point of 0.02 because, although arbitrary, we believed that it provided the most reasonable trade-off between high performance and computational burden. Specifically, as can be seen visually from figures in the Learning Curve Results section, this stopping point typically marks the beginning of the “point of diminishing returns,” where the power law curves begin to plateau, and the amount of additional sample needed to make further improvements increases exponentially. It is important to note that once the learning curve equations are estimated, this choice of stopping point can be freely altered. Therefore, although we report 0.02 in this study as our stopping rule of interest, the final equations derived below can be re-estimated with any user-specified stopping point (for example, 0.01 or 0.05). We present sample size results using alternative thresholds of 0.01 and 0.05 AUC points from the full–dataset AUC in Multimedia Appendix 1.

Once the raw data was generated, estimated learning curves were fit using nonlinear least squares optimization [28], following the power law equation: AUC*_(_*_n_*_)_* = an^b^+c, where a and b were estimated, and c was either fixed to be the full–dataset AUC or was also estimated, depending on the quality of the fit. For some datasets and algorithms, the power law function did not fit the data well. These were typically scenarios where the dataset required a relatively larger sample size to become stable. In these scenarios, we instead fit the learning curves using a logarithmic function, AUC_(n)_=β_0_+β_1_*log(n), where β_0_ and β_1_ were estimated using ordinary least-squares [29].

### Sample Size Determination and Guidelines

Following the learning curve analysis of the 4 selected algorithms on our datasets, we examined the effects of 6 dataset–level factors on the sample sizes needed for AUC stability. These included minority class proportion (maximum value of 50%, indicating no class imbalance), separability (defined as the full–dataset AUC itself), the total number of features, the percentage of features that were continuous (versus binary or discrete numeric), the percentage of “core linear” features, and “dataset nonlinearity.” Core linear features were determined by adding an L1 (LASSO [least absolute shrinkage and selection operator]) penalty to the LR model for each full dataset [30]. The percentage of variables that did not shrink to zero when this penalty was added were defined as core linear features. Dataset nonlinearity was a rough measure of the degree of nonlinear or interactive relationships between the predictors and the outcomes that were present in the data. This was defined as the point difference in the full–dataset AUC when using a complex algorithm (XGB) compared with LR. For example, if LR yielded a full–dataset AUC of 0.90 and XGB yielded a full–dataset AUC of 0.95, the dataset nonlinearity would be calculated as 5.0. For the purpose of calculating these values, XGB hyperparameters were left at their default values [26].

Within the context of each algorithm, the relationship between these dataset–level variables and the n required for AUC stability was examined. Since the estimated sample sizes were discrete and right-skewed numeric values, we used negative binomial regression models [31] to quantify the strength and significance of each dataset characteristic on predicted sample sizes, which produce coefficients in terms of log-expected counts. Then, in multivariable negative binomial regression models for each algorithm, we selected up to 3 dataset–level predictors that together minimized the Akaike Information Criterion, which evaluates how well the model fits the data while penalizing for the number of parameters estimated [32]. A maximum of 3 predictors per model were considered in order to avoid potential overfitting.

We also calculated adjusted deviance-based pseudo-*R*^2^ statistics [33,34], which further quantified each model’s goodness-of-fit and proportion of deviance explained by the predictors. The final model equations were reported and discussed for each algorithm, and visualizations of the model predictions at varying levels of each dataset-level characteristic were generated. Statistical significance was set to α=.05 for all hypothesis tests considered, and RStudio (version 4.2.3; R Foundation for Statistical Computing) was used for all analyses.

## Results

**Figure 1:**
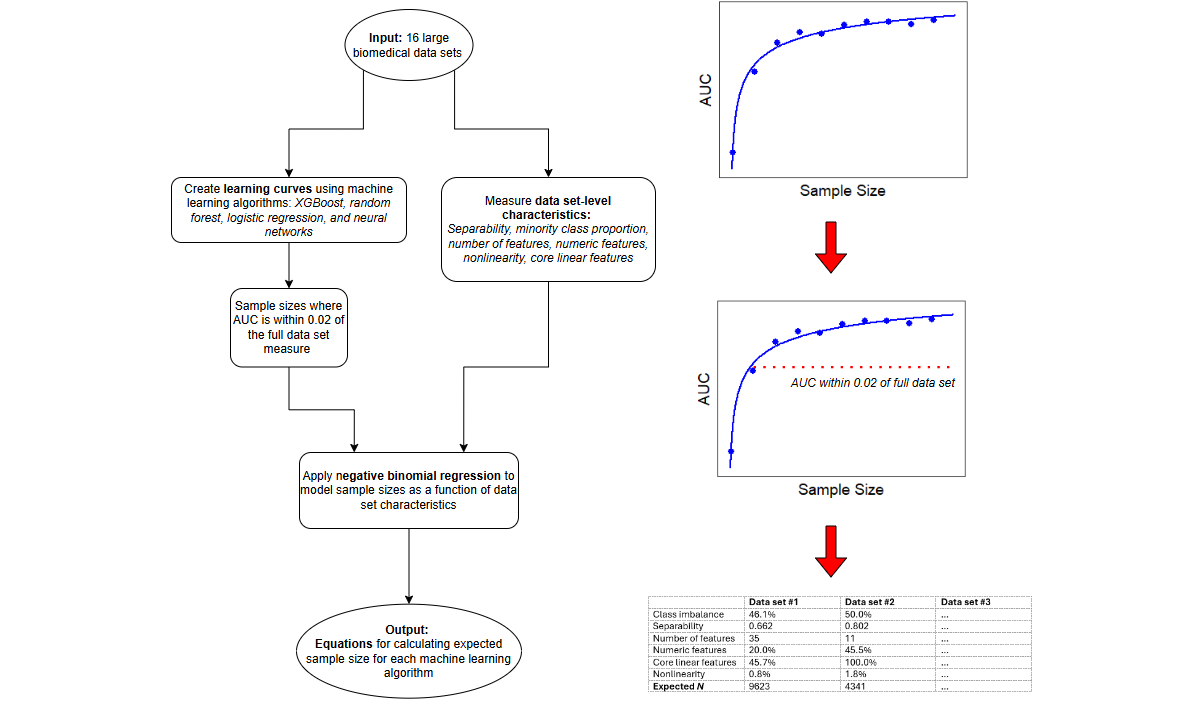
Flowchart of study aims and deliverables. AUC: area under the curve.

A workflow and overview of the study’s aims and end points can be seen in Figure 1.

We gathered 16 datasets with sample sizes ranging from 70,000 to 1,000,000 (Table 1). Out of the 4 classification algorithms examined, XGB performed the best or tied for the best performance on 14/16 (87.5%) datasets, while RF performed the best on two. Full dataset AUCs (separability) ranged from 0.608-0.979 (XGB), 0.609-0.976 (RF), 0.596-0.949 (LR), and 0.603-0.974 (NN; Table 2). As expected, LR models generally performed the worst, with full–dataset AUCs that were 0.028 points lower on average compared with XGB.

**Table 1:** Dataset–level characteristics.

Dataset name	Source	Full dataset size, n	Minority class proportion (%)	Features, n	Core linear features (%)	Continuous features (%)	Nonlinearity
Cardio	OpenML	70,000	50	11	100	45.5	1.8
Diabetes130	OpenML	101,766	46.1	35	45.7	20	0.8
NoShow	OpenML	110,527	20.2	8	25	12.5	1.2
BreastTumor	OpenML	116,640	34.6	9	44.4	33.3	9.5
Diabetes	UCI	253,680	13.9	21	85.7	19	0.7
COVID-19	OpenML	263,007	39	16	37.5	6.25	2.2
LOS	OpenML	318,438	2.1	11	36.4	27.3	1.9
CDC Heart Disease (2020)	Kaggle	319,795	4.4	17	76.5	23.5	0.5
CDC Heart Disease (2022)	Kaggle	394,509	8.6	39	59	15.4	0.6
Heart	OpenML	1,000,000	44.4	13	92.3	46.2	1.6
Hepatitis	OpenML	1,000,000	20.8	19	94.7	31.6	4.0
Lymph	OpenML	1,000,000	45.7	18	83.3	5.6	2.3
Pharynx	OpenML	1,000,000	25.6	11	72.2	18.2	1.5
Cholesterol	OpenML	1,000,000	16.5	13	61.5	30.8	6.7
Dermatology	OpenML	1,000,000	13.2	33	84.8	3	3.6
PBC	OpenML	1,000,000	17.8	18	83.3	55.6	6.2

**Table 2:** Full–dataset AUC (separability) for each algorithm.

Dataset name	XGB^a^	RF^b^	LR^c^	NN^d^
Cardio	0.802	0.796	0.784	0.795
Diabetes130	0.662	0.661	0.654	0.661
NoShow	0.608	0.609	0.596	0.603
BreastTumor	0.777	0.780	0.682	0.730
Diabetes	0.829	0.822	0.822	0.826
COVID-19	0.664	0.661	0.642	0.661
LOS	0.917	0.915	0.898	0.901
CDC Heart Disease (2020)	0.815	0.810	0.810	0.809
CDC Heart Disease (2022)	0.815	0.801	0.809	0.801
Heart	0.965	0.963	0.949	0.962
Hepatitis	0.979	0.976	0.939	0.974
Lymph	0.957	0.957	0.934	0.956
Pharynx	0.858	0.858	0.843	0.856
Cholesterol	0.736	0.728	0.669	0.714
Dermatology	0.859	0.857	0.823	0.852
PBC	0.850	0.850	0.788	0.823

^a^XGB: XGBoost.

^b^RF: random forest.

^c^LR: logistic regression.

^d^NN: neural network.

### Learning Curve Results

**Figure 2:**
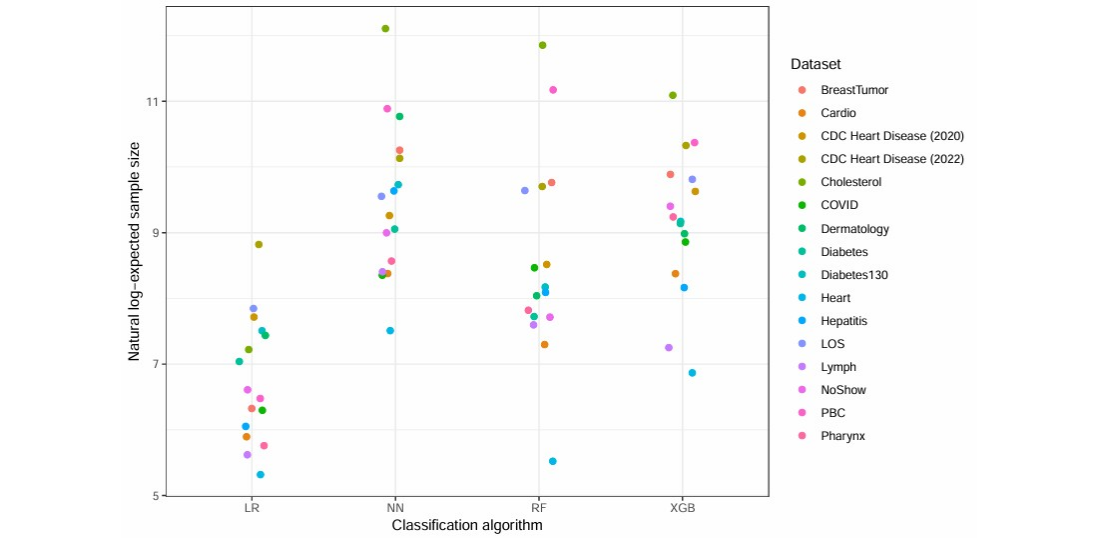
Visualization of expected sample sizes calculated from the learning-curve analysis of 16 data sets. LR: Logistic Regression. NN: Neural Networks. RF: Random Forest. XGB: XGBoost.

**Figure 3:**
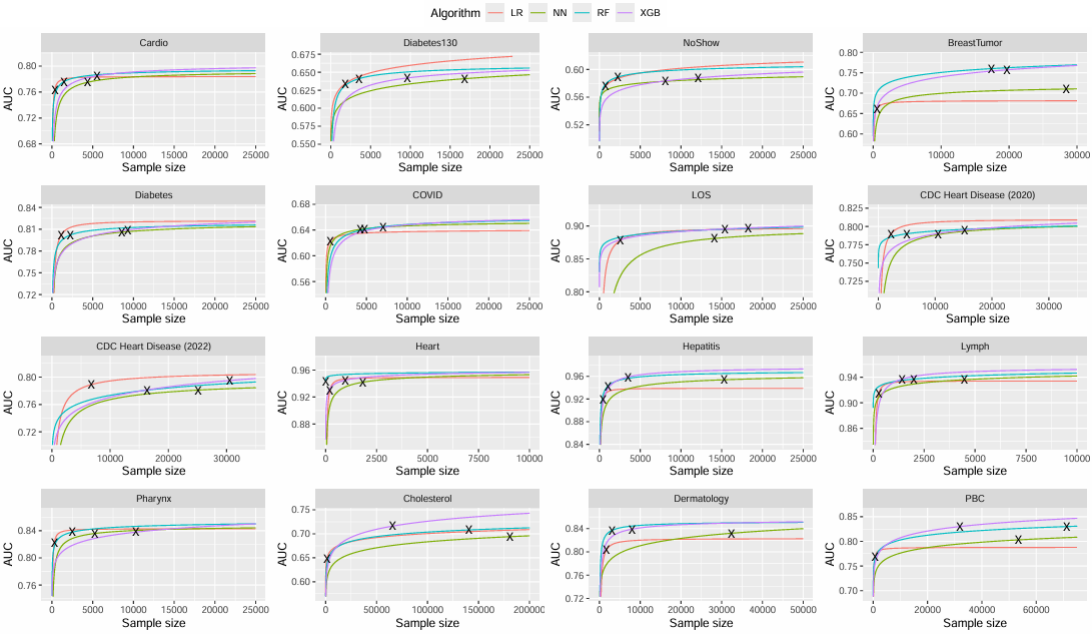
Fitted learning curves for 16 data sets across 4 classification algorithms. Different colors were chosen for different algorithms Each black "X" represents the point where the AUC at size n first comes within 2-points (or, 0.02) of the asymptotic (ie full-data set) AUC. LR: Logistic Regression. NN: Neural Networks. RF: Random Forest. XGB: XGBoost. AUC: Area-under the receiver operating characteristic curve.

Learning curves were fit to the 16 collected datasets. Table 3 and Figure 2 contain a full summary and visualization of estimated sample sizes across each classification algorithm and dataset. NNs required the largest sample sizes to reach stability and also had the most variability among the datasets (median 12,298, range 1824-180,835). LR required the smallest sample size to reach stability and also was the least variable (median 696, range 204-6798). XGB required approximately 3 times the sample size compared with RF, but the range of estimated sample sizes generated from RF models was nearly twice as wide (Table 2). Figure 3 shows the fitted learning curves for each algorithm generated within each dataset, with a marker indicating the earliest sample size where the CV-AUC was within 2 points of the full–dataset AUC.

**Table 3:** Sample sizes are needed to reach AUC stability from the learning curve analysis.

Dataset name	XGB^a^	RF^b^	LR^c^	NN^d^
Cardio, n	4341	1476	363	4349
Diabetes130, n	9623	3544	1822	16,823
NoShow, n	12,114	2241	742	8084
BreastTumor, n	19,668	17,383	558	28,424
Diabetes, n	9306	2261	1140	8556
COVID-19, n	7026	4750	543	4241
LOS, n	18,239	15,381	2555	14,085
CDC Heart Disease (2020), n	15,177	4995	2243	10,510
CDC Heart Disease (2022), n	30,534	16,355	6768	25,120
Heart, n	960	250	204	1824
Hepatitis, n	3513	3265	425	15,302
Lymph, n	1409	1992	276	4470
Pharynx, n	10,296	2488	317	5260
Cholesterol, n	65,556	140,499	1368	180,835
Dermatology, n	7979	3103	1696	47,489
PBC, n	31,897	71,194	650	53,453
Median (Range)	9960 (960-65,556)	3404 (250-140,499)	696 (204-6798)	12,298 (1824-180,835)
Mean (SD), Log-Transform	9.16 (1.11)	8.57 (1.55)	6.75 (0.96)	9.47 (1.17)

^a^XGB: XGBoost.

^b^RF: random forest.

^c^LR: logistic regression.

^d^NN: neural network.

### Dataset–Level Characteristics

**Figure 4:**
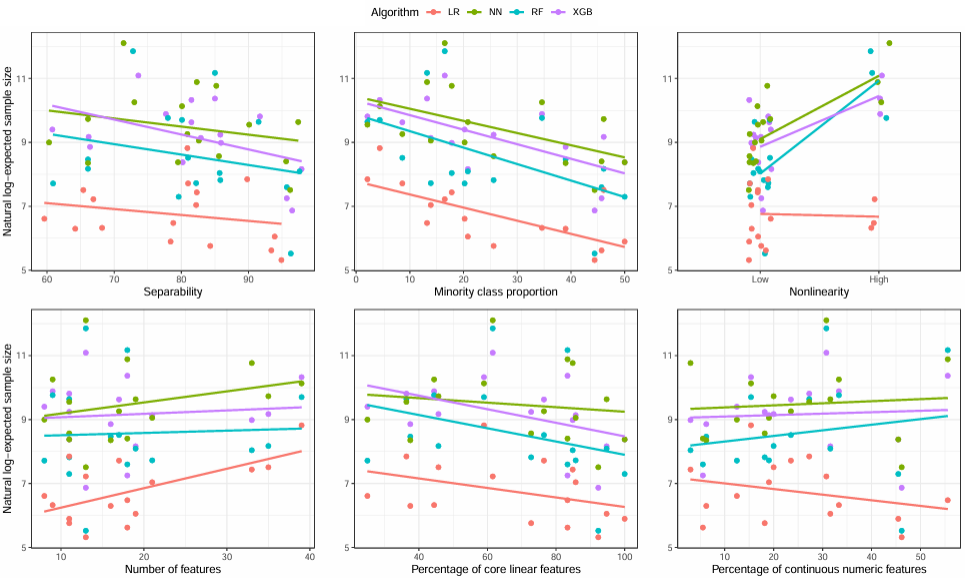
Relationships between each data set–level characteristic and expected sample sizes. The y-axis represents the natural-log transformed sample size values, while each x-axis represents varying levels of each data set–level characteristic. Separability multiplied by 100. All values representing proportions were multiplied by 100 so that 0 indicates 0% and 100 indicates 100%. Nonlinearity “low”: <5, “high”: ≥5. LR: Logistic Regression. NN: Neural Networks. RF: Random Forest. XGB: XGBoost.

Dataset–level characteristics were examined (Table 1). The average minority class percentage was 25.18% (SD 15.93%), and the average number of features was 18 (SD 9). The average percentage of continuous numeric features was 24.6% (SD 15.31%), and the average percentage of core linear features was 67.69% (SD 23.73%). The median dataset nonlinearity was 1.85 (range 0.50-9.50). Most datasets (13/16, 81.3%) had nonlinearity values under 5.0. Thus, for the purpose of model fitting, this was converted into a binary variable indicating either "high" (≥) or "low" (<5) nonlinearity. Scatterplots examining the visual relationships between log-expected sample sizes and each dataset–level characteristic can be found in Figure 4.

Negative binomial regression models were fitted, examining the individual associations between each of the dataset–level characteristics and predicted sample sizes (Table 4). In these models, separability (full–dataset AUC) was multiplied by 100 for easier interpretation. For example, an AUC of 0.80 was entered as 80.0 in the models. For XGB, minority class proportion and separability were both inversely related to sample size; for every 1-unit increase in separability (where 50.0 was the baseline value), estimated sample sizes were affected by a multiplier of 0.955 (*P*=.02). For every 1% increase in minority class proportion, estimated sample sizes were affected by a multiplier of 0.959 (*P*<.001). In datasets with high (≥5.0) values of nonlinearity, estimated sample sizes were affected by a multiplier of 3.888 (*P*=.005). In the RF analyses, results were similar for minority class proportion (0.931× multiplier for 1% increase, *P*<.001), separability (0.939× multiplier for each 1-unit increase over 50.0, *P*=.047), and nonlinearity (15.984× multiplier for those with high values, *P*<.001). However, the percentage of continuous numeric features (1.065× multiplier for every 1% increase, *P*=.003) was also individually statistically significant. For LR, minority class proportion (0.963× multiplier for every 1% increase, *P*=.001), the number of features (1.056× multiplier for each additional feature, *P*=.006), the percentage of core features (0.982× multiplier for 1% increase, *P*=.046), and the percentage of continuous numeric features (0.971× multiplier for 1% increase, *P*=.04) were significantly associated with sample size. Again, a more balanced ratio of classes reduced the needed sample size, while more features increased the sample size. However, a higher percentage of core linear features and a higher percentage of continuous numeric features lowered the sample size. Finally, for NNs, results were similar to XGB; minority class proportion (0.953× multiplier for 1% increase, *P*=.003), full–dataset AUC (0.950× multiplier for each 1% increase over 50.0, *P*=.03), and nonlinearity (6.85× multiplier for high values, *P*<.001) were all individually statistically significant.

**Table 4:** Univariable association of each dataset–level characteristics with predicted sample size.

Variable	XGB^a^			RF^b^			LR^c^				NN^d^		
	Estimate (95% Confidence Interval)	*P* value	Estimate (95% Confidence Interval)		*P* value	Estimate (95% Confidence Interval)		*P* value	Estimate (95% Confidence Interval)				*P* value
Minority class proportion	0.959 (0.934-0.985)	<.001	0.931 (0.891-0.977)		<.001	0.963 (0.945-0.983)		.001	0.953 (0.921-0.990)				.003
Separability	0.955 (0.903-1.000)	.02	0.939 (0.861-1.028)		.047	0.999 (0.946-1.051)		.98				0.950 (0.898-1.007)	.03
Number of features	1.000 (0.958-1.053)	.99	0.966 (0.904-1.059)		.35	1.056 (1.021-1.097)		.006				1.001 (0.947-1.071)	.98
Continuous features (%)	1.019 (0.986-1.057)	.21	1.065 (1.013-1.121)		.003	0.971 (0.942-1.006)		.04	1.019 (0.982-1.059)				.04
Core linear Features (%)	0.983 (0.958-1.001)	.07	0.988 (0.941-1.034)		.41	0.982 (0.959-1.004)		.046	0.994 (0.960-1.028)				.61
Dataset nonlinearity	3.889 (1.631-11.209)	.005	15.985 (5.914-55.754)		<.001	0.585 (0.211-2.112)		.35	6.853 (2.763-20.947)				<.001

^a^XGB: XGBoost.

^b^RF: random forest.

^c^LR: logistic regression.

^d^NN: neural network.

In multivariable models for each algorithm, we selected the set of 3 predictors that minimized the AIC. The equation below, as well as Table 5, presents a summary of each algorithm-specific model, which shows the adjusted contribution of each predictor to the expected sample size.

Equation 1: Empirically derived sample size equations for XGB, RF, LR, and NN algorithms.

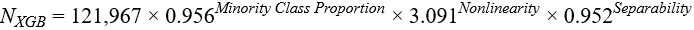

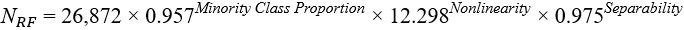

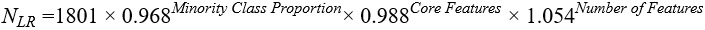

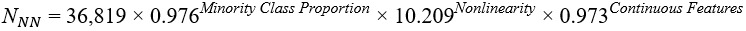

**Table 5:** Multivariable negative binomial regression—data-level characteristics effect on predicted sample size.

Variable	XGB^a^				RF^b^					LR^c^					NN^d^		
	Estimate (95% Confidence Interval)		*P* value		Estimate (95% Confidence Interval)		*P* value		Estimate (95% Confidence Interval)			*P* value		Estimate (95% Confidence Interval)			*P* value
Intercept	121,967 (57,883-262,108)		<.001		26,872 (8118-92,378)		<.001		1801 (904-3,809)			<.001		36,819 (18,691-76,970)			<.001
Minority class proportion	0.956 (0.946-0.967)	<.001		0.957 (0.940-0.975)		<.001		0.968 (0.957-0.979)			<.001		0.976 (0.957-0.996)			.02	
Separability	0.952 (0.934-0.970)		<.001		0.975 (0.947-1.004)		.07		—^e^			—		—			—
Number of features	—		—		—		—		1.054 (1.034-1.076)			<.001		—			—
Continuous features (%)	—		—		—		—		—			—		0.973 (0.950-0.997)			.02
Core linear features (%)	—		—		—		—		0.988 (0.980-0.996)			.005		—			—
Dataset nonlinearity	3.091 (2.011-4.922)		<.001		12.298 (5.826-28.791),		<.001		—			—		10.209 (4.274-26.569)			<.001

^a^XGB: XGBoost; adjusted pseudo-*R*^2^=0.845.

^b^RF: random forest; adjusted pseudo-*R*^2^=0.808.

^c^LR: logistic regression; adjusted pseudo-*R*^2^=0.798.

^d^NN: neural network; adjusted pseudo-*R*^2^=0.665.

^e^Not available.

**Figure 5:**
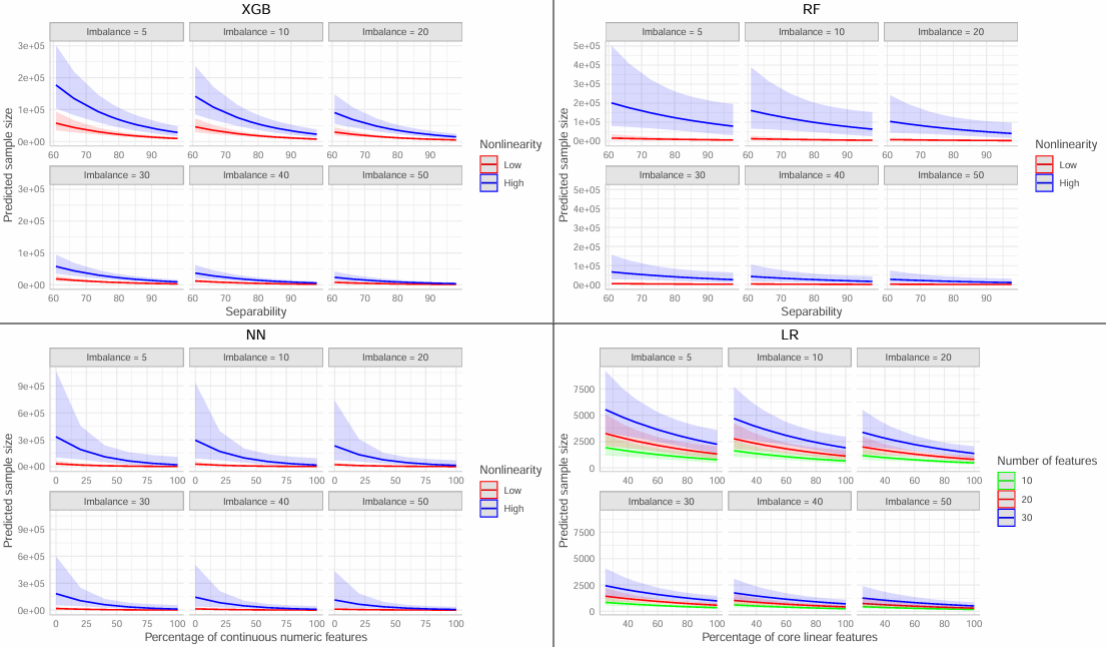
Fitted values derived from the 4 final negative binomial regression models for each classification algorithm. Shaded lines represent 95% CI. Imbalance = minority class proportion × 100. Separability = Full–data set AUC × 100. Nonlinearity “low”: <5, “high”: ≥5. LR: Logistic Regression. NN: Neural Networks. RF: Random Forest. XGB: XGBoost.

For XGB and RF, minority class proportion, separability, and nonlinearity were the top 3 variables selected. For LR, minority class proportion, number of features, and percentage of core features were the top 3 variables. For NN, the top 3 variables were minority class proportion, number of features, and nonlinearity. The direction and magnitude of coefficient estimates from multivariable models were similar to those obtained from univariable models (Table 5). Deviance-based *R*^2^ statistics, adjusted for the number of predictors added, were 0.845 (XGB), 0.808 (RF), 0.798 (LR), and 0.665 (NN; Table 5). This indicated that the dataset–level predictors explained a majority (66.5%-84.5%) of the total deviance in the data among all 4 models, although the NN model was weaker than the other 3. Figure 5 shows the predicted sample sizes estimated from each algorithm-specific model at a variety of levels for each predictor. As can be seen, for all 4 classification algorithms, a balanced class ratio (50% cases versus 50% controls) resulted in the lowest predicted sample sizes.

## Discussion

### Conclusions

In this study, we performed a learning curve analysis of 16 datasets over 4 different classification algorithms. From this, we identified the expected samples needed to reach AUCs within 2 points of those measured in the full dataset. We then examined the effects of dataset–level characteristics on expected sample sizes and provided formulas that can be used to predict the necessary sample size in a new dataset. We found that LR required the smallest sample size (median 696, range 204-6798) but performed slightly worse, on average, compared with more complicated algorithms. RF (median 3404, range 250-140,499) and XGB algorithms (median 9960, range 960-65,556) required larger sample sizes, as expected. NNs required the largest sample size (median 12,998, IQR 1824-180,835) and also had the most variability over the 16 datasets. This was an interesting finding, as our implementation of a deep learning approach was a basic architecture including only one hidden layer and 20 nodes. The fact that NNs required the largest median sample size of >12,000 and were the most variable in terms of expected sample size demonstrates that implementation of deep learning methods—especially more complex deep-learning schemes than what we have provided—should probably be reserved for extremely large datasets for optimal performance and adequate discriminative stability. In addition, NNs had a weaker performance than XGB in every dataset. However, it is possible that a more expressive deep learning approach may yield higher AUCs compared with the best-performing ML solutions considered in this study, although to the best of our knowledge, no other studies have provided deep learning results that outperform ML approaches in these specific datasets. These results support current literature that suggests deep learning may not be optimal for tabular data analysis compared with tree-based methods [35] when weighing accuracy trade-offs versus computational burden.

Our results are consistent with Perlich et al [12], which showed that LR might be optimal in small samples, but tree-based methods eventually provide the best performance in large datasets. Van der Ploeg et al [16] determined that LR required a much lower number of events-per-variable for AUC stability, defined as CV-AUC within 0.01 of the full–dataset performance compared to RF and NN, which required >200 events-per-variable. We can convert our expected sample sizes to events-per-variable by taking the predicted n, multiplying it by the minority class proportion of the dataset, and then dividing it by the number of features. In our study, LR required an average of 11 events-per-variable, XGB: 205, RF: 231, and NN: 342, which supports this notion that modern modeling techniques are “data-hungry” [16].

In summary, this study provides a simple framework for determining sample size in the context of 4 popular ML algorithms. Dataset–level variables that altered expected sample sizes varied by algorithm, but the class imbalance of the outcome, the strength and number of features, and the nonlinearity of the predictors were among the most influential characteristics. Most of these dataset–level characteristics can be reasonably guessed or influenced before the study begins. For example, researchers can examine previous studies in their field of interest to determine a reasonable range for separability and minority class proportion. For minority class proportion, which was a key selected feature for all 4 models, we observed that an optimal class balance (50% cases, 50% controls) led to the lowest predicted sample sizes, with each additional percentage point of balance decreasing the needed n by a multiplier of 0.96-0.98.

In addition, researchers can use feature engineering to control the quality and overall number of predictors included in their models. As we have determined in this study, a smaller number of strong predictors will generally require less sample size than a large and noisy predictor set, supporting the idea that more features are not always ideal [36]. Dataset nonlinearity is less intuitive to guess before data collection. In general, we found that datasets with nonlinearity values of at least 5.0 required approximately 3-12 times the amount of sample to reach stability, depending on the algorithm. However, in this study, 13/16 (81.3%) of the datasets had values under 5.0, which means that high values of dataset nonlinearity may be uncommon. Again, previous studies where both simple (LR) and complex (NN, RF, and XGB) methods are compared can help researchers determine if this value will be high. As a last resort, researchers can simply calculate expected sample sizes for both scenarios (<5.0 and ≥5.0) using the model equations presented in this study and discuss the implications. It is also important to note that the effect of nonlinearity (and other dataset–level characteristics) on estimated sample sizes is diminished when the class imbalance is optimized. This is due to the multiplicative nature of the negative binomial regression models, which is illustrated in Figure 5. Thus, first and foremost, it is critical that researchers aim to collect a sample with the most balance between cases and controls.

To our knowledge, no previous study has presented specific formulas for calculating sample size within the context of ML using multiple dataset–level characteristics. Although other studies have provided estimates of the needed sample size (or number of events per variable) to reach performance stability over a variety of classification algorithms, these works used simulation approaches or a limited number of real-life datasets and did not consider multiple specific dataset–level characteristics in calculation of these estimates [15,16].

### Limitations and Future Work

One limitation of our study was the relatively small sample size of only 16 datasets to develop our final models. Although this number is a relatively large amount in this area of research—similar learning curve analyses have typically examined less than 10 [14,16,17]—assessment of more would strengthen these models and provide clearer insight into dataset–level effects on expected sample size. However, the fact that we still observed many statistically significant relationships even with this small effective sample size is a strength of the study. In addition, the datasets we examined were all tabular in nature and had relatively low (<50) numbers of features—generalization of the formulas presented in this study may not extrapolate to datasets with larger amounts of features or data arising from medical imaging or nontabular sources. Finally, it is important to note that ML, specifically algorithms like RF and XGB, can still outperform traditional parametric methods even if the sample size is limited (ie, under n=5000) and when hyperparameter tuning is implemented [37,38]. Therefore, these guidelines should serve as a supplement to be used in the first stage of predictive modeling, giving a general idea of how much sample is expected to reach a point of “diminishing returns,” where large amounts of additional data will only increase the AUC marginally.

Future research in this area could examine different outcome types, such as regression, multiclass, or survival end points—or different performance metrics, such as area under the precision-recall curve or Brier score for probability calibration [10]. In addition, a more in-depth examination of XGB, RF, and NN hyperparameters would be impactful, as all of the equations developed in this study considered only the default hyperparameter values, which could limit the generalizability of the results. However, in practice, it would be extremely difficult to guess plausible values of hyperparameters before data collection, so examination of different configurations would mostly be educational in nature and impractical to incorporate in sample size equations before any data collection. Finally, stacked ML methods [39], or different gradient-boosted tree algorithms such as CatBoost [40] or LightGBM [41] could be investigated.

## Appendix

Multimedia Appendix 1 Additional information: data processing steps and additional tables and figures.

## Abbreviations

AUC: area under the curve
CV: cross-validation
CV-AUC: cross-validated area under the curve
LASSO: least absolute shrinkage and selection operator
LR: logistic regression
MCAR: missing completely at random
ML: machine learning
MLP: multilayer perceptron
NN: neural network
RF: random forest
XGB: XGBoost

## Data Availability

The datasets generated during and/or analyzed during this study are publicly available and can be accessed using links found in Multimedia Appendix 1. In addition, the datasets generated during and/or analyzed during this study are available from the corresponding author on reasonable request. An RShiny app is publicly available where researchers can calculate the expected sample size based on our formulas with user-input dataset-level characteristics and can be found at [42].
